# Metal-organic frameworks as biosensors for luminescence-based detection and imaging

**DOI:** 10.1098/rsfs.2016.0027

**Published:** 2016-08-06

**Authors:** Sophie E. Miller, Michelle H. Teplensky, Peyman Z. Moghadam, David Fairen-Jimenez

**Affiliations:** Department of Chemical Engineering and Biotechnology, University of Cambridge, Pembroke Street, Cambridge CB2 3RA, UK

**Keywords:** metal-organic frameworks, biosensors, luminescence, contrast agent

## Abstract

Metal-organic frameworks (MOFs), formed by the self-assembly of metal centres or clusters and organic linkers, possess many key structural and chemical features that have enabled them to be used in sensing platforms for a variety of environmentally, chemically and biomedically relevant compounds. In particular, their high porosity, large surface area, tuneable chemical composition, high degree of crystallinity, and potential for post-synthetic modification for molecular recognition make MOFs promising candidates for biosensing applications. In this review, we separate our discussion of MOF biosensors into two categories: quantitative sensing, focusing specifically on luminescence-based sensors for the direct measurement of a specific analyte, and qualitative sensing, where we describe MOFs used for fluorescence microscopy and as magnetic resonance imaging contrast agents. We highlight several key publications in each of these areas, concluding that MOFs present an exciting, versatile new platform for biosensing applications and imaging, and we expect to see their usage grow as the field progresses.

## 1. Introduction

The use of biosensors to detect, and in some cases quantify, the presence of a compound has been a prominent area of research. A biosensor is defined as a device with biological sensing elements connected to or integrated within a transducer [1–4]. Their classification has grown since the initial work from Clark & Lyons in 1962 [5]. Sensors using polymers [1,6], nanoparticles [7,8] and enzymes [5,9] have all emerged since the field began, among various others [10,11]. Porous materials such as zeolites and, more recently, metal-organic frameworks (MOFs) [12] possess several advantageous properties for sensing applications. Having attracted a significant amount of attention in fields such as catalysis [13–18], gas storage and separation [19–21], ion exchange [22–24], and more [16,25,26], these materials are notable for their well-defined high porosity and large surfaces areas for adsorption [27].

MOFs, which are formed by the self-assembly of metal ions or clusters and organic linkers, have been explored extensively as chemical sensors [27–29]. The most common MOF sensing platform takes advantage of the inherent luminescence of many frameworks. Luminescent MOF sensors have been successfully implemented for the detection of oxygen [30,31], explosive chemicals [32,33], various aromatic compounds [34] and amines [35,36], yet limited examples exist for biological sensing applications [37]. MOFs' key structural and chemical features make them appealing candidates for measuring levels of biochemical compounds or for imaging contrast agents. Several different MOFs exhibit low cytotoxicity, which is important for eventual *in vivo* applications [38]. Furthermore, the wide range of metal and organic building blocks that can be incorporated into their structures allows for judicious tuning of the materials' light absorption and emission properties to avoid interference with those of the desired analyte, in addition to design for specific affinity. MOFs can also be modified post-synthesis, enabling specific molecular recognition [39]. For biosensing applications, this versatility opens up possibilities for cell- or tissue-specific targeting [26]. Finally, the MOF can be re-used multiple times, as the binding and fluorescence events are typically reversible.

**Figure 1.**
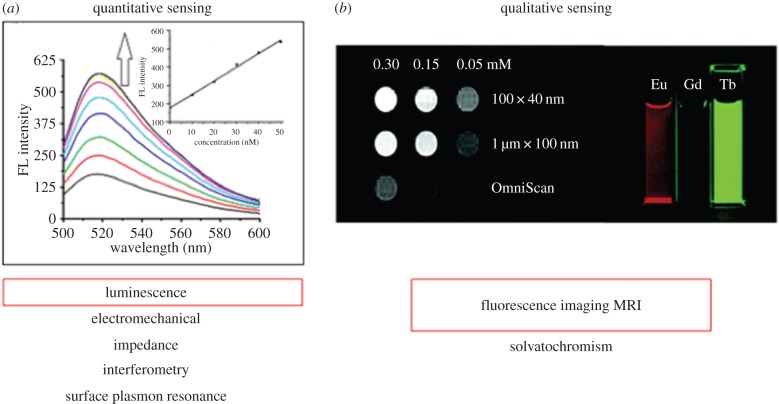
Overview of different sensing platforms and specific ones to be discussed in this review. (*a*) Fluorescence spectra showing proportional increase of signal of HIV-DNA interacting with MOF [40]. (*b*) T_1_-weighted MRI of gadolinium-based MOF effectively enhancing water signal compared to clinically used OmniScan as well as dispersions of doped MOF nanorods highly luminescing [41].

Herein, we present a review of the literature on MOFs as biosensors. We split our analysis into two key areas: quantitative and qualitative sensing (figure 1). Quantitative sensing involves the selective interaction of an analyte with a MOF and the use of inherent or post-synthetically imparted MOF luminescence for visual observation of the sensing event and direct measurement in solution or *in vitro* [42]. Mechanical and electrical schemes have not been included in this review so as to focus on the luminescence element of biosensing. Qualitative sensing, on the other hand, involves the use of MOF luminescence to locate and visualize a cellular region of interest using optical microscopy. Qualitative sensing also comprises the use of MOFs as contrast agents for confocal microscopy or magnetic resonance imaging (MRI), which are useful techniques for biomedical imaging and diagnosis.

## 2. Quantitative luminescence-based sensing

MOF fluorescence often arises from the linker molecule, as the aromatic or other conjugated linkers of many MOFs absorb light in the UV–visible range. While these MOFs typically contain d^10^ metal ions, which do not exhibit light-emitting electronic transitions, MOFs with f-element metal centres (e.g. lanthanide and actinide metals) have also been used as luminescent MOF sensors. The molecular structure of these luminescent MOFs further enhances their sensing ability; the highly absorbing conjugated linkers amplify emission throughout the extended network as energy is transferred from node to node [27]. Luminescence sensing with MOFs can be achieved through the enhancement, quenching or shifting of fluorescence signals upon target adsorption.

### 2.1. Lanthanide-based metal-organic frameworks for biomolecule detection

One of the first instances of MOFs for luminescence sensing was reported by Rieter *et al*. [43], who synthesized a nanoscale MOF (NMOF) that was further modified for sensing of dipicolinic acid (DPA). DPA is the main component of bacterial endospores such as *Bacillus anthracis*, which was used as the agent for anthrax attacks, and is present in the endospore casing, making up to 15% of its mass [43,44]. Therefore, the detection of these spores through DPA sensing would be important in preventing bioterrorism attacks. While many techniques have been used to detect DPA, including spectrophotometry [45], Raman spectroscopy [46], pyrolysis mass spectrometry [47], high-performance liquid chromatography [48], potentiometric sensing [49] and capillary zone electrophoresis [50], these suffer from high costs, the need for large instruments, and time-consuming and complicated analysis. Nanoparticle-based sensors are promising as smaller, faster, simpler, and therefore more efficient alternatives to these methods.

To this end, Rieter *et al*. [43] synthesized a silica-coated NMOF Ln(BDC)_1.5_(H_2_O)_2_, where Ln represents a lanthanide metal (Eu^3+^, Gd^3+^ or Tb^3+^) and BDC stands for 1,4-benzenedicarboxylate. Ln-MOFs are particularly interesting as luminescent sensors because of their visible, pure colour and their relatively long luminescence lifetimes resulting from f–f transitions [51]. For luminescence sensing of DPA, Eu-doped Gd(BDC)_1.5_(H_2_O)_2_@SiO_2_ nanoparticles were further modified with Tb-ethylenediaminetetraacetic acid (EDTA) monoamide, which was covalently attached to the silica shell. Tb^3+^ ions and molecular Tb complexes have been used for luminescence sensing of DPA [52–55], as DPA forms emissive complexes with these groups. These Tb-EDTA-modified nanoparticle NMOFs were able to sense DPA at concentrations as low as 48 nM in ethanolic solution. Without DPA, there is minimal, non-interfering emission with excitation at 278 nm attributed to Eu, which thus acts as an internal calibration. Upon addition of DPA at low concentrations, signal intensity increased in a linear fashion, levelling off at concentrations above 50 µM. These results are comparable with those of other nanoparticle sensing systems; for example, molecularly imprinted metal nanoparticles have wider linear regimes extending to concentrations of 120 µM but higher detection limits (100 nM) [56,57], while polymeric nanoparticles can have detection limits as low as 10 pM but a linear relationship between DPA concentration and fluorescence up to only 100 nM [58]. However, MOFs' simple, self-assembly-based synthesis and versatile post-synthetic modification make them particularly attractive over these other materials. Furthermore, these NMOFs were shown to be selective for DPA over other biologically relevant molecules, such as the amino acid l-alanine, in a quantitative assay in a tris(hydroxymethyl)aminomethane (Tris) buffered ethanol-in-water solution.

**Figure 2.**
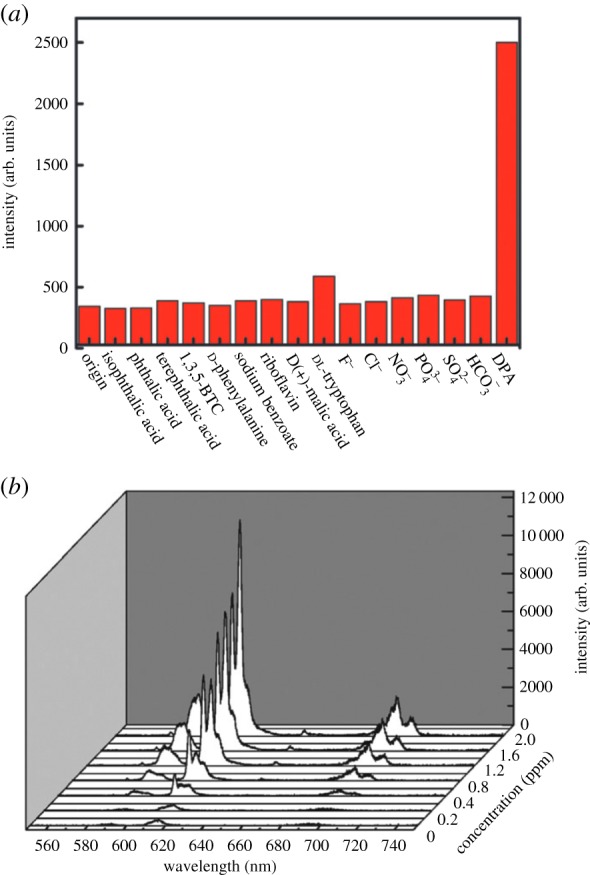
(*a*) Signal intensities of NMOF-1 with 1.0 ppm of different analytes (excited at 279 nm). Specificity for DPA is demonstrated. (*b*) Photoluminescence spectra of a solution of NMOF-1 with the addition of different concentrations of DPA, indicating a correlation [59].

One drawback of this example, however, is the need for multiple steps of post-synthetic modification to achieve the desired effect. Ideally, the MOF would be used directly to fully take advantage of its unique, versatile chemical and structural properties. To this end, Xu *et al.* [59] developed another fluorescence-based sensor for DPA using the NMOF Eu_2_(FMA)_2_(OX)(H_2_O)_4_*4H_2_O (NMOF-1, where FMA is fumarate and OX is oxalate). It was observed that the addition of small amounts of DPA significantly enhances the inherent fluorescence of NMOF-1 at 590, 617 and 698 nm in ethanol. These peaks are associated with different characteristic electronic transitions of Eu^3+^ ions. DPA molecules are hypothesized to interact with Eu^3+^ ions in a way that enhances intramolecular transfer and thus increases the MOF's fluorescence. Luminescence intensity was linearly proportional to the amount of DPA in solution, and the MOFs were selective for DPA over several other molecules also found in bacterial endospores, including different aromatic di- and tricarboxylic acids, amino acids and various metal ions (figure 2). Additionally, the nanoscale particle size led to improved detection of DPA, with the NMOF showing 90 times greater fluorescence intensity in the presence of 2 ppm DPA compared to bulk MOF-1 due to the greater surface area in solution for interaction between the DPA and metal ions in the MOF.

### 2.2. Peroxidase-mimic metal-organic framework biosensors

In another class of MOF biosensors, the MOF catalyses a luminescence-producing reaction involving the target species. In particular, several MOFs have shown peroxidase-like activity, capable of catalysing oxidation reactions in the presence of H_2_O_2_. The iron-containing MIL-series MOFs (named after the Materials Institute Lavoisier) have been studied by several groups as biomimetic catalysts to produce colorimetric assays for detecting different compounds. Ai *et al*. [60] showed that solvothermally synthesized MIL-53 is capable of oxidizing 3,3′,5,5′-tetramethylbenzidine (TMB), *o*-phenylenediamine (OPD) and 1,2,3-trihydroxybenzene in the presence of H_2_O_2_, producing a deep-blue colour similar to that observed for the horseradish peroxidase enzyme. To elucidate the catalytic mechanism, photoluminescence measurements were performed that indicated that MIL-53 could be involved in an electron transfer process to produce OH• radicals from H_2_O_2_, which in turn react with the molecule being oxidized. This same general mechanism likely governs the activity of all of the peroxidase-like MOF sensors discussed here, and is consistent with the chemistry of the horseradish peroxidase, which also uses H_2_O_2_ as an oxidizing agent and has an iron-containing haeme cofactor in its active site.

The actual intact MOF is crucial in producing the catalytic effect, as it was determined that Fe^3+^ ions did not leach from MIL-53 and react with the species in solution. MIL-53 obeyed Michaelis–Menten kinetics, and the *K*_M_ obtained with H_2_O_2_ as the substrate (0.04 mM) was much lower than that for horseradish peroxidase (6.36 mM) [61] and other peroxidase-mimic materials (i.e. Fe_3_O_4_ nanoparticles or nanocomposites, gold nanoparticles, CeO_2_ nanoparticles and iron-containing mesoporous silica nanoparticles), indicating a stronger affinity for the H_2_O_2_ substrate [62–66]. More recent work has shown that a microwave-assisted synthesis of MIL-53 can further lower *K*_M_ values (0.03 mM and 0.28 mM for H_2_O_2_ and TMB, respectively) by yielding small, purely octahedral crystals [67].

These authors then observed that ascorbic acid (AA), a common and biomedically relevant water-soluble vitamin, inhibits oxidation of OPD by MIL-53, enabling a ‘turn-off’ colorimetric assay for AA. AA was detected by measuring the decrease in absorption at 450 nm produced by the oxidation of OPD by the OH• radicals. A linear relationship is observed between AA concentration and absorption in the range of 28.6–190.5 µM, with a lower detection limit of 15 µM. However, this quenching-based ‘turn-off’ method is less favourable than ‘turn-on’ mechanisms, as events other than analyte binding can diminish fluorescence and reduce accuracy [27]. To confirm selective quenching by AA over other species, future studies would be needed. Furthermore, while the sensor is successful in detecting AA, other existing sensors are markedly superior, with orders of magnitude larger linear ranges and lower detection limits [68,69]. Therefore, for AA sensing, these peroxidase-like MOF biosensors will require significant improvements to compete with other technologies.

Instead of sensing AA, Dong *et al.* used MIL-53 to develop a sensor for glucose detection [67]. Glucose oxidase, which catalyses the oxidation of glucose to produce H_2_O_2_, was added to the MIL-53 and TMB system. As described above, the H_2_O_2_ that is formed reacts with TMB in the presence of the MOF to produce the measurable colour change. Notably, the *K*_M_ values obtained for H_2_O_2_ reduction were at least seven times lower than those for MIL-88(Fe)-NH_2_ [70] and iron porphyrin-modified MOF hemin@MIL-53(Al)-NH_2_ [71]. The reduced *K*_M_ values indicate increased sensitivity towards the desired substrate. Electron spin resonance experiments supported the previous hypothesis that the MIL-53 decomposes H_2_O_2_ into OH• radicals, which then attack TMB to produce the observed colour change. The sensor detected glucose with a lower limit of 0.25 mM and a linear range of 0.25–20 mM, and was used successfully in real human serum samples. The MOFs also showed high selectivity for glucose over other sugars common in blood serum, exhibiting nearly identical performance in a mixture containing maltose, lactose and fructose.

Another MOF, MIL-101(Fe), possesses several advantageous material properties for sensing applications, including large pore sizes (2.9 and 3.4 nm, with windows of 1.2 and 1.6 nm) [72], high surface area per unit mass (5500 m^2^ g^−1^) [73], stability in air and water [42], accessible exposed metal sites, and organic ligands for functionalization. Furthermore, it has a small band gap that enables excitation by visible light; yet, despite these characteristics, MIL-101(Fe) has not been as widely explored as a peroxidase-mimic for colorimetric biosensing. Recently, Cui and co-workers [42] prepared a catalyst for H_2_O_2_ reduction by growing crystals of Prussian blue (Fe_7_(CN)_18_, PB) onto their MIL-101(Fe) MOF. PB consists of cyanide molecules coordinated to an iron ion centre, forming a high surface area crystalline coordination polymer with peroxidase-like catalytic properties [74–76]. However, its poor dispersibility, difficulty to functionalize and strongly interfering blue colour make it difficult to use on its own in liquid solution colorimetric biosensing. The MIL-101, therefore, provides a solid support with large surface area and coordinating Fe^3+^ ions on which uniform PB nanostructures with high catalytic activity can be formed. Additionally, this hybrid structure takes advantage of MIL-101(Fe)'s large porosity for analyte adsorption and PB's high electroactivity, which results from its regular crystal structure and homogeneous distribution of electron and ion charge transfer rates, for improved catalysis [77].

Cui *et al.* [42] demonstrated the ability of their PB/MIL-101(Fe) MOFs to catalyse the oxidation of TMB, OPD and 2,2′-azinobis(3-ethylbenzthiazoline-6-sulfonate) in the presence of H_2_O_2_, which formed reactive OH• radicals that further react to produce a visible colour change. Kinetics were shown to be linear with respect to H_2_O_2_ concentration in the range of 2.40–100 mM, with a detection limit of 0.15 mM, comparable with other MOFs [60,78]. Similar Michaelis–Menten kinetics were observed as discussed above, with the PB/MIL-101(Fe) showing lower *K*_M_ values for TMB and H_2_O_2_ than the MOF alone (0.127 and 0.0580 mM, compared with 0.490 and 0.620 mM, respectively). To improve the biocompatibility, stability and targeting ability of the particles *in vitro* and *in vivo*, the MOF's surface was modified with silica, 3-aminopropyltriethoxysilane, polyethylene glycol (PEG) or folic acid (FA). These reagents adsorbed to iron atoms and carboxyl groups, which compromised catalytic activity as a result of blocked active sites. In particular, the addition of FA enabled colorimetric detection of cancer cells with upregulated FA receptors (MCF-7), as the PB/MIL-101(Fe)-FA bound much more strongly to the cell surface than PB/MIL-101(Fe) without FA, and showed increased absorbance with the number of MCF-7 cells [42].

Another well-known MOF, HKUST-1, composed of unsaturated copper ion nodes and 1,3,5-benzenetricarboxylic acid struts, was employed as a catalyst for the oxidation of luminol by H_2_O_2_ to produce the reaction's characteristic chemoluminescence [79]. The luminol-H_2_O_2_-HKUST-1 system could be used as a ‘turn-off’ sensor for the detection of dopamine (DA). While the luminol–H_2_O_2_ reaction typically occurs quite slowly under basic conditions, with HKUST-1 the luminescence intensity produced by the reaction increased about 90-fold. It is hypothesized that the HKUST-1 facilitates electron transfer and radical generation on its surface, with fluorescence and electron paramagnetic resonance spectra confirming that the amount of OH• generated from H_2_O_2_ increased with the addition of more HKUST-1. UV–visible and luminescence spectra were used to elucidate the reaction mechanism, which was identical to the typical mechanism in which luminol reacts sequentially with two OH• radicals to produce the final luminescent product. Thus, HKUST-1 acts as a non-reactive catalyst in the reaction. It is expected that the high surface area-to-volume ratio and electron density of HKUST-1 result in greatly enhanced luminescence relative to Cu^2+^ alone in solution.

For the detection of DA using HKUST-1, luminescence intensity scaled linearly with DA concentration in the range of 0.010–0.70 M, with a detection limit of 2.3 nM (signal : noise ratio S/N = 3). When used in human plasma and urine samples, other common chemical compounds including various ions, amino acids, sugars and vitamins were shown not to interfere with DA sensing, resulting in less than 5% deviation of the signal. Such low signal interference makes this ‘turn-off’ sensor comparable to typically more robust ‘turn-on’ sensors.

These peroxidase-like MOF biosensors present exciting possibilities, using H_2_O_2_ reduction as a basis for colorimetric or luminescence-based assays for the detection of a wide range of species, from molecules to cells. These materials showcase the impressive versatility of MOFs, which can themselves act as catalysts or can be used for the immobilization of other compounds that enhance activity and functionality. Furthermore, mimicking the capabilities of biological enzymes using man-made materials is a powerful tool that can enable the diversification of chemical processes achievable at the laboratory and, eventually, industrial scales.

### 2.3. Metal-organic framework biosensors with nucleic acid probes

**Figure 3.**
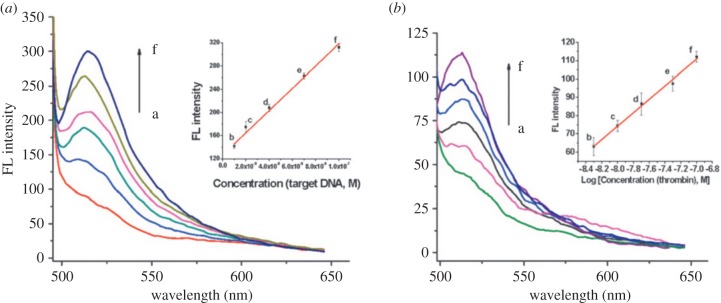
(*a*) Fluorescence spectra of the FAM-labelled DNA–Cu(H_2_dtoa) in the presence of different concentrations of target DNA. Inset: plot of fluorescence intensity versus concentrations of target DNA. (*b*) Fluorescence spectra of the FAM-labelled probe DNA 2–Cu(H_2_dtoa) in the presence of different concentrations of thrombin. Inset: plot of fluorescence intensity versus logarithm of concentrations of thrombin. The concentration of dye-labelled probe DNA 1 and DNA 2 is 50 nM [80].

In addition to sensing of small molecules, MOFs have also been used to detect larger biomolecules such as DNA or proteins. The two-dimensional MOF N,N^′^-bis(2-hydroxyethyl)dithiooxamidatocopper(II) [Cu(H_2_dtoa)] was used for targeting of the HIV-1 U5 long terminal repeat sequence, as well as of thrombin, an enzyme that participates in blood coagulation and clotting, with high sensitivity and selectivity [80]. Its Cu^2+^ coordination centres have intrinsic fluorescence quenching properties, while the dithiooxamide linkers' conjugated π-electron systems enable non-covalent binding of single stranded DNA (ssDNA) molecules. The binding of fluorophore (carboxyfluorescein, or FAM)-conjugated probe ssDNA via π-stacking interactions with the MOF will quench its fluorescence via photoinduced electron transfer (PET) to Cu^2+^. When the target ssDNA is added, the probe is then released from the framework, restoring fluorescence and allowing for the ‘turn-on’ sensing of the viral gene (figure 3). Upon the addition of target DNA (50 nM) and thrombin (100 nM, also expected to interact with the probe DNA), fluorescence intensities recover 156% and 502%, respectively. The sensor showed impressive selectivity to both targets, with single-base mismatched DNA showing minimal change in fluorescence, and in the case of thrombin, no effect of T6 lysozome, bovine serum albumin, and human IgG enzymes on signal. The linear ranges for DNA and thrombin detection were 10–100 nM and 5–100 nM with detection limits of 3 nM and 1.3 nM, respectively. These values are comparable with those of carbon nanotube (1.8–14.5 nM) and graphene oxide platforms (2.0–5.0 nM) [81–86].

The same MOF, Cu(H_2_dtoa), has also been used to develop a sensor for *in vitro* sequence-specific sensing of double-stranded DNA (dsDNA) [87]. Detection of dsDNA is important because traditional mechanisms of sensing DNA require the inconvenient generation of ssDNA to bind with a complementary sequence [88]. Cu(H_2_dtoa) was selected here for its strong chemisorption of a FAM-labelled triplex-forming oligonucleotide (TFO) probe and its fluorescence quenching properties. Fluorescence is recovered when TFO is released from the MOF after interaction with the target dsDNA, a 16-bp polypurine tract sequence of HIV-1 RNA, via hydrogen bonding with the dsDNA's major groove. The fluorescence signal scales linearly with dsDNA concentration over the range of 4–200 nM, and has a low detection limit of 1.3 nM (S/N = 3), an order of magnitude more sensitive than graphene oxide-based or electrochemical DNA sensors, which have achieved detection limits of 14.3 and 10 nM, respectively [88,89]. The sensor performance was reproducible between several parallel experiments, and exhibited high sequence selectivity for the target dsDNA over dsDNA with one or two base pair mutations, as well as ssDNA complementary to the probe.

A similar strategy was pursued by Yang and co-workers [40] to develop a sensor for HIV-1 dsDNA and Sudan virus (an ebolavirus) RNA sequences. Here, a water-stable three-dimensional MOF with Cu^2+^ nodes and a combination of two linkers, *N*-carboxymethyl-3,5-dicarboxylpyridinium bromide and 4,4′-dipyridyl sulfide, was modified with target-specific FAM-labelled probe DNA. The net positive surface charge of the MOF particles and the aromatic groups on both linkers enable electrostatic and hydrogen-bonding/π-stacking interactions of the probe DNAs with the MOF, as well as PET-induced fluorescence quenching. Fluorescence anisotropy measurements confirmed that the probe DNA interacts more strongly with the target DNA and RNA sequences than with the MOF, which would enable it to be released from the MOF and bind to the target sequences in solution, where its fluorescence would no longer be quenched.

This system demonstrated a linear relationship between target concentration and fluorescence up to 50 nM for both sequences, with detection limits of 196 pM and 73 pM for the viral dsDNA and RNA, respectively. The selectivity of the probes for the desired targets was confirmed, with recovery of fluorescence much less pronounced when mutated and non-specific sequences were added to the assay. Non-target sequences exhibited diminished effect of concentration on fluorescence, and resulted in between 50 and 86% less fluorescence than the target sequence in the dsDNA assay, and between 67% and 96% less fluorescence in the RNA assay [40]. The authors also tested the performance of the MOF biosensors with longer lengths of target Sudan virus RNA sequences, and discovered that the assay was able to detect RNA sequences between 20 and 80 bp in length. This finding could open doors towards development of similar sensors using MOFs with larger pores and longer probe DNA sequences.

In addition to the sensing of nucleic acid sequences, MOFs functionalized with FAM-labelled ssDNA have been used for the detection of mercury(II) ions in solution [90]. The use of MOFs to detect ions is a particularly promising area of research within the field of MOF sensors. Several groups have reported the use of lanthanide-based MOFs (Ln-MOFs) for highly sensitive detection of ions such as Cu^2+^ [51], Fe^3+^ [91,92], many lanthanides [93] and various other cations [51] and anions [94]. The cations typically interact with Lewis basic linker molecules in the Ln-MOF frameworks, whose luminescence results from the electronic properties of the lanthanide ions in the framework and changes in response to adsorbed compounds. Anion sensing, on the other hand, was achieved by doping an Al-based MIL-121 MOF with lanthanide cations [94]. Another group demonstrated a post-synthetically modified zinc-based MOF capable of selectively sensing toxic cyanide ions [95].

**Figure 4.**
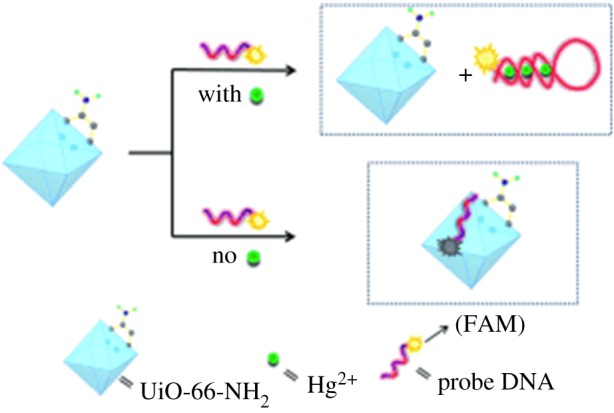
A MOF/DNA hybrid system, where UiO-66-NH_2_ is modified with thymine-rich FAM-labelled ssDNA. Hg^2+^ is known to bind specifically to T–T mismatched base pairs, forming a T–Hg^2+^–T ‘sandwich complex’ when in solution. This, therefore, works to detect Hg^2+^ sensitively and selectively [90].

Sensing of mercury, in particular, is important because mercury is harmful to both human health and the environment, as it is highly soluble in water and even at low concentrations is known to adversely affect the endocrine, nervous and excretory systems [96]. Furthermore, existing detection methods are time consuming and require sophisticated instruments [97–99]. The platform developed by Wu *et al*. uses Zr-based Universitetet i Oslo (UiO)-66-NH_2_ modified with thymine-rich FAM-labelled ssDNA, as Hg^2+^ is known to bind specifically to T–T mismatched base pairs by forming a T–Hg^2+^–T ‘sandwich complex’ (figure 4) [100–102]. Similar to the previous examples above, the ssDNA interacts with the MOF via hydrogen bonding and π–π stacking interactions between the DNA bases and the aromatic moieties on the organic linker molecules. The amine group of the linker acts as an ‘anchor’ on the MOF surface, and fluorescence of the FAM group is quenched via PET with 75% efficiency. Upon the addition of Hg^2+^ to the ssDNA-modified MOF sensing system in Tris–HCl buffer (pH 7.4), fluorescence increases more than twofold. The fluorescence of the sensor increases rapidly at low concentrations of Hg^2+^, slowing as more is added, with the linear range observed between 0.1 and 10 µM, and a detection limit of 17.6 nM, which is below the WHO-reported toxicity level of 30 nM in water [103]. The detection limit is furthermore comparable with other technologies using aptamers to sense mercury, with reported detection limits ranging from 0.6 nM to 1 µM [104–108], as well as other non-aptamer-based sensing platforms using carbon nanotubes (14.5 nM) [83], metal nanoparticles (32 nM) [109], macrocyclic cyclodextrin molecules (10 nM) [110] or porphyrin (8 nM) [111]. The sensor also shows considerable selectivity over other metal ions (Ca^2+^, Cd^2+^, Co^2+^, Cu^2+^, Fe^2+^, Fe^3+^, Mg^2+^, Mn^2+^, Ni^2+^, Pb^2+^), which only showed slight response in comparison with the significant fluorescence increase in the presence of Hg^2+^ (*F*/*F*_o_ is approximately 2.75 for Hg^2+^, but in the range of 0.5–1.5 for the others).

With a diverse array of targets, from ions to small molecules to nucleic acid sequences, luminescence-based MOF sensors have proved to be a powerful platform for quantitative biosensing applications. These assays allow for simple and direct quantification of the species being detected using fluorescence emission measurements, and the materials are relatively easy to fabricate with the potential for scale-up.

## 3. Qualitative sensing: optical and magnetic resonance imaging contrast agents

MOFs also demonstrate utility as sensors in a qualitative manner. Imaging and the ability to demonstrate the presence of certain molecules, proteins, receptors or cell types have been recently explored [112,113]. The use of MOFs as sensors in this capacity often requires the use of a targeting molecule, sometimes requiring the addition of a coating layer for optimal functionalization [114–116] and sometimes not [117–119]. There are two main modes of qualitative sensing: the use of MOFs as optical agents, and the use of MOFs as MRI contrast agents. Optical agents, more simplistic than their counterparts, are discussed first with zinc, zirconium and iron-carboxylate-based examples.

### 3.1. Utilization of metal-organic framework luminescence for fluorescence imaging

Liu *et al.* [120] implemented MOFs as an agent for optical imaging. Trying to avoid self-quenching due to large Stokes shifts, biodegradable phosphorescent MOFs using Zn and Zr metal clusters were created for *in vitro* use. The MOFs were synthesized to create the phosphorescent Ru complex {Ru[5,5′-(CO_2_H)_2_-bpy}(bpy)_2_)(PF_6_)_2_, where bpy is 2,2′-bipyridine. A 2-day synthesis procedure with this ligand and Zn(NO_3_)_2_ produced the product, which achieved a dye loading of 78.7%. These particles, due to their instability in water and other biologically relevant media, were unsuccessfully coated with a silica layer. Therefore, a more stable MOF was formulated using a zirconium metal cluster, and these particles were used in optical imaging capacities. The particles, spherical in shape and on average 85 nm in diameter, were able to enter cells through mechanisms of endocytosis [121]. Testing of the particle stability in various solvents demonstrated decomposition in 8 mM phosphate buffered saline (PBS), a commonly used biologically relevant medium, thus a thin shell of silica added increased the half-life (*t*_1/2_) by approximately 2.7 h. The half-life increase was deemed enough to perform optical imaging studies, although whether the time scale allowed for cellular uptake of the various particle types was unstated by the authors. The silica coating additionally provided silanol groups that were further modified with PEG, and in some cases, anisamide-bonded PEG. Anisamide (also referred to as AA, but for clarity here referred to as AA*), a targeting ligand, has moderate affinity for sigma receptors, which are overexpressed in various tumour cells. When attached to particles, anisamide has been shown to increase the particles' intracellular delivery into prostate and lung cancer cells [115,120,122]. Demonstration of the synthesized compound's ability to function as an optical imaging agent involves ensuring maintained cell viability. MTS assays (3-(4,5-dimethylthiazol-2-yl)-5-(3-carboxymethoxyphenyl)-2-(4-sulfophenyl)-2H-tetrazolium compound used in a colorimetric assay to assess cell metabolic activity) were conducted with concentrations of these particles ranging from 0 to 100 µg well^−1^ and showed negligible decrease in cell viability over the range and in a 24 h incubation time. *In vitro* efficacy as a contrast agent demonstrated enhanced uptake of anisamide-targeted particles, shown by both laser scanning confocal fluorescence microscopy and ion coupled plasma mass spectrometry (ICP-MS) analysis of Ru content in lysed cell pellets.

Taylor-Pashow *et al.* [116] used a modified version of the MIL-101 MOF with iron carboxylate to load a fluorophore along with an anti-cancer drug. The synthesized MOF had lower porosity most likely due to structural defects, and an average size of 200 nm. The original MOF, Fe_3_(μ_3_-O)Cl(H_2_O)_2_(BDC)_3_, was altered using amino-functionalized BDC ligands. This ensured an easy mechanism for post-synthetic modifications through covalent attachment. In this instance, 1,3,5,7-tetramethyl-4,4-difluoro-8-bromomethyl-4-bora-3a,4a-diaza-s-indacene (Br-BODIPY) was reacted to attach the BODIPY component (an optical imaging contrast agent) covalently to the MOF, achieving around 6–12 wt% loadings. The benefit about tracking this particular molecule in confocal microscopy is the quenching element: BODIPY-grafted MOF is non-emissive due to luminescence quenching by the d–d transitions of the Fe(III) centres, meaning that only during release of the molecule will the signal be obtained. This differs from the mechanism of Liu *et al.* [120], where one of the MOF components itself (metal cluster or ligand) is the fluorescent marker for imaging. The release of BODIPY from the MOF was quantified to provide a *t*_1/2_ of approximately 2.5 h in 8 mM PBS at 37°C. More characterization experiments could be performed to claim more confidently that this is due to the degradation of the particles because of the covalent amine linkage, as opposed to adsorption of the dye onto the MOF. A thin silica layer was applied to the MOFs to slow down the release of cargo and create novel core–shell nanostructures. This increased *t*_1/2_ to approximately 16 h in PBS buffer at 37°C. Optical imaging was done using laser scanning confocal microscopy with HT-29 human colon adenocarcinoma cells. Fluorescent labelling was demonstrated in a dose-dependent manner, with the largest dose of 0.38 mg ml^−1^ of nanoparticles showing the largest level of fluorescence inside the cells. This indicated a successful MOF uptake and notable optical imaging using a cargo-loaded iron-based MOF.

Nishiyabu *et al.* [123,124] explored the incorporation of various different dyes into the nanoparticle structures. These amorphous particles were self-assembled from nucleotides and lanthanide ions in water—specifically gadolinium. Water-soluble dyes were used to allow for simultaneous inclusion during particle formation. Spectroscopy revealed the amount of dye loaded to the structure, and, due to the coordination of the dyes's carboxyl groups to the Gd^3+^ ions, binding of anionic dyes was more efficient. Confocal laser scanning microscopy performed with NADH/La^3+^ microspheres doped with a dye demonstrated dye internalization in the particle. The same technique was also used to demonstrate cell internationalization of 5′-AMP/Gd^3+^ dye-doped nanoparticles co-localized to lysosomes. This was done through the use of LysoTracker Red, a hydrophobic fluorescence probe used to determine the location of lysosomes. Owing to the success of cellular uptake, an *in vivo* study was performed to study tissue localization of dye-doped 5′-AMP/Gd^3+^ nanoparticles. In this case, a form of optical imaging called fluorescence reflectance imaging was used in addition to the quantitative measure of ICP-MS. The results demonstrated potential for these dye-doped nucleotide/lanthanide nanoparticles to be imaging agents for the liver due to the high level of accumulation in this organ. However, this rapid clearance is most likely due to undesirable recognition from the hepatic reticuloendothelial system [125], and thus future applications from this work are limited.

### 3.2. Metal-organic frameworks as magnetic resonance imaging contrast agents

Contrasting agents have also been used in MRI [113,115,126], where normal tissues are separated from diseased tissues based on varied nuclear magnetic resonance water proton signals that come from different densities and/or nuclear relaxation rates [41,127,128]. Contrast agents are incredibly useful for enhancing this form of non-invasive diagnostics [129]. Current methods of MRI, however, require administration of high doses of contrast agents to counteract the low sensitivity of the technique [126]. Owing to this, the use of MOFs has been incorporated to lower the required dose of agent necessary and capitalize on the quantity of metal clusters located within the structure.

As for the metal used in practice, applications of Gd^3+^ have been common to increase water proton relaxation rates [41]. Gadolinium is clinically accepted and thus has been the foundation for multimodal imaging for multiple studies in the literature [41,130,131]. Rieter *et al.* [41], in particular, formulated nanorods with Gd metal clusters and BDC ligands. It was hypothesized that the degradation of the particles causes the release of numerous metal centres and thus gives large relaxivities on a per particle basis. As a variety of sizes of Gd(BDC)_1.5_(H_2_O)_2_ were created, an inverse size dependence of per millimolar Gd^3+^ relaxivity was observed—consistent with the decreasing surface-to-volume ratio. These *r*_1_ (longitudinal relaxivity) and *r*_2_ (transverse relaxivity) values range from 20.1 and 45.7 mM^−1^ s^−1^ for the largest nanorods (approx. 1 µm in length by approx. 100 nm in diameter) to 35.8 and 55.6 mM^−1^ s^−1^ for the smaller nanorods (approx. 100 nm in length by approx. 40 nm in diameter), respectively. It was postulated that metal centres close to the exterior surface of the MOF were responsible for changes in relaxivities. These initial experiments compared water signal intensities of T_1_-weighted MOF-added samples with a commercial agent: OmniScan. Higher efficiency in water signal enhancement was found when the MOF was added, while for T_2_-weighted spin-echo pulse sequence images, an increase in water signal perturbation was observed more so in comparison to OmniScan. Thus, this Gd-based MOF is acceptable as both a T_1_ and T_2_ contrast agent depending on the MR pulse sequence employed.

However, even though gadolinium is clinically accepted, it has been described as problematic for MRI future use, due to potential leaching concerns associated with the metal ion—responsible for the fatal nephrogenic systemic fibrosis condition [115]. A number of studies in the literature show different metal clusters being applied in the MOF synthesis protocols including manganese [115,126] and iron [113]. These metals have demonstrated lower cytotoxicity in all studies indicating a new direction of agent-based ions used. Additionally, if successful, the MOF nanoparticles can be dual-utilized as a diagnostic and a drug-delivery device—a form of multimodal imaging.

**Figure 5.**
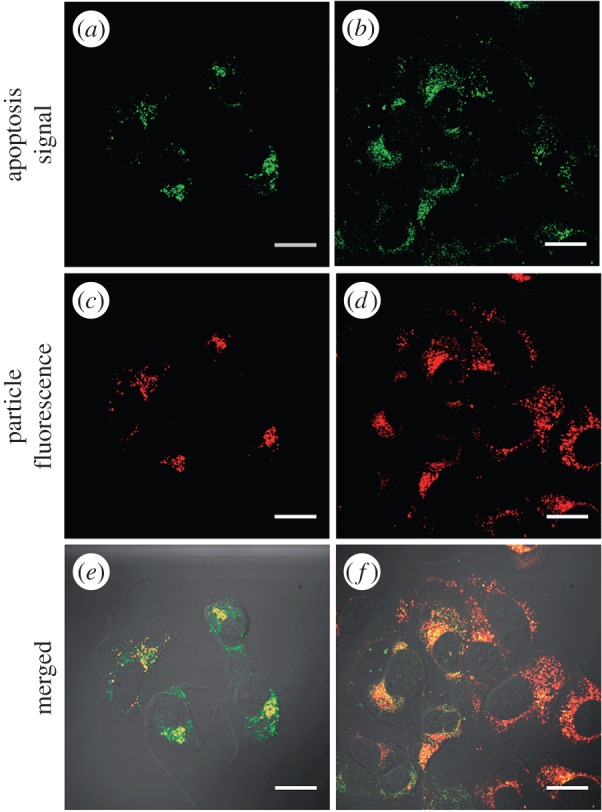
Confocal laser scanning microscopy images of MCF-7 cells incubated with untargeted PEGylated chlorin e6-doped Mn-bisphosphonate MOF (*a*,*c*,*e*) and AA*-functionalized PEGylated chlorin e6-doped Mn-bisphosphonate MOF (*b*,*d*,*f*). Channels are Annexin V FITC conjugate early apoptosis stain (green) and chlorin e6 from the modified particles (red). Scale bars are 20 µm [115].

Liu *et al.* [115] used synthesized multifunctional MOFs with loadings of zoledronate—an effective nitrogen-containing bisphosphonate—and targeting components to demonstrate that this theranostic platform could deliver a therapeutic accurately while acting as an MRI contrast agent. A Mn-bisphosphonate MOF was formulated with a microwave reaction and amorphous particles were created. While this is not ideal for utilization of pore space for cargo loading in other MOF applications, it sufficed for the demonstration of particle targeting, efficacy and MRI. The synthesized particles contained approximately 63 wt% zoledronate loading, but rapidly decomposed in the presence of 5 mM PBS in less than 1 h. A lipid/lipid-PEG layer was added to alter the release kinetics, achieving a nanoparticle about 20 nm larger in diameter. The loading of drug was then determined again to be lower at 42 wt%, potentially due to the release of cargo in the PEGylation process. The targeting of the particle was done with anisamide, used previously by Liu *et al.* [120], to target the same sigma receptors in H460 cells. AA* was added covalently onto the PEG-grafted layer in a 10 mol% incorporation. No significant morphological differences between the AA*-functionalized PEG-grafted and untargeted PEG-grafted counterpart were noted. In this instance, MCF-7 human breast adenocarcinoma cells and AsPC-1 human pancreatic cancer cells were the cell lines used. Cytotoxicity studies with MCF-7 of targeted and untargeted MOFs (IC_50_ = 2.0 µM, 6.4 µM, respectively) compared with free zoledronic acid groups and unPEGylated MOFs demonstrated a significant decrease in cell viability as measured through the trypan blue exclusion assay. Additionally, a 48 h incubation with AsPC-1 cells showed a similar trend in IC_50_values (IC_50_ = 13 µM for targeted and 24 µM for untargeted). Confocal microscopy was also used to visually demonstrate the targeting effectiveness on cancer cell mortality. However, this was unsuccessful due to lack of intrinsic fluorescence of the MOF. Therefore, the synthesis protocol was modified to allow for doping with chlorin e6, a red dye, of 2 wt%. Lipid coating, PEGylation and AA* markers were all added, and the co-localization of the signal with the LysoTracker green signal demonstrated that the particle was uptaken by MCF-7 cells to a higher degree than untargeted particles (figure 5). The efficacy of the particle was partially successful to an unknown efficiency, as demonstrated by confocal microscopy and flow cytometry results. Confocal microscopy images showed higher presence of Annexin V-FITC in the targeted particle. Annexin V-FITC was added to mark apoptosis by binding with phosphotidyl serines expressed on the external membrane by cells [132]. Approximately 70% compared with 86% of cells were undergoing some stage of apoptosis in the targeted versus untargeted, respectively. All this displays one of two modalities of the multifunctional system.

**Figure 6.**
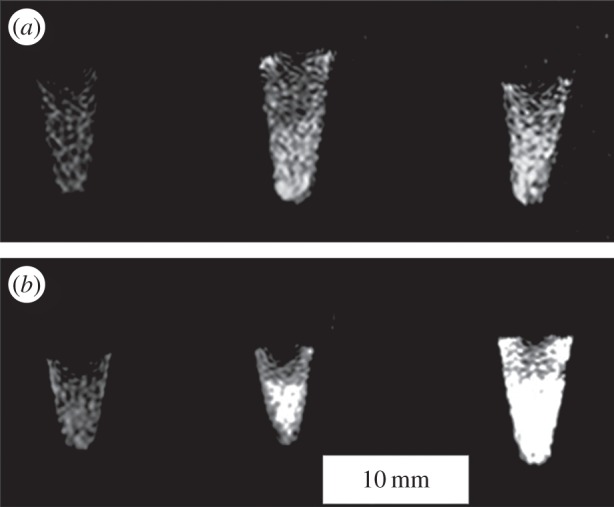
T_1_-weighted images of MCF-7 cell pellets: (left) cells incubated without any nanoparticles, (middle) cells incubated with PEGylated Mn-bisphosphonate MOF and (right) cells incubated with AA*-functionalized PEGylated Mn-bisphosphonate MOF at 8 h (*a*) and 24 h (*b*) time point [115].

Studies using this manganese-based system for diagnostic MRI applications determined three different sets of relaxivity rates for *r*_1_ and *r*_2_ with a 3 T scanner. The MOF alone had *r*_1_ = 4.66 mM^−1^ s^−1^ and *r*_2_ = 22.2 mM^−1^ s^−1^. The untargeted but PEGylated MOF had *r*_1_ = 11.6 mM^−1^ s^−1^ and *r*_2_ = 19.7 mM^−1^ s^−1^. The targeted MOF had *r*_1_ = 7.6 mM^−1^ s^−1^ and *r*_2_ = 70.3 mM^−1^ s^−1^. These values follow the logical explanations proposed by the authors of the system, namely that the metal cluster centres of the AA*-functionalized particles had a reduced influence on the water environment because of the AA* hydrophobicity. These targeted *r*_1_values, however, are on a par with, or higher than, most of those reported in the literature, which are around 0.1–7 mM^−1^ s^−1^ [133–136]. *In vitro* studies showed large levels of contrast with T_1_-weighted images for the AA*-functionalized particles with decreasing contrast for untargeted particles and no particles, respectively (figure 6). Uptake studies involving ICP-MS supplied Mn^2+^ content for each system, at 191.3 µg g^−1^ cell, 99.1 µg g^−1^ cell and approximately 50 µg g^−1^ cell, respectively. The MRI system was able to demonstrate a greater degree of contrast with a lower amount of contrast agent added compared with current values, which are several grams per person [41].

Other uses of manganese-based MOFs for MRI rely on the disintegration of the particles to greatly spike the contrast potential due to a high number of Mn^2+^ ions [126]. MOFs were synthesized using reverse-phase microemulsions established by Rieter *et al.* [41] and two different linkers, BDC and trimesic acid (BTC). The formulated nanorods ranged from diameters of 50 to 100 nm and lengths of 750 nm to 2 µm, suggesting that some of these particles might have been too large for cellular uptake [121]. Microwave-synthesized MOFs with BTC ligands changed morphologies such that a block-like MOF was formed with 50–300 nm lengths in the three dimensions. These particles were further altered for MRI cellular imaging through addition of a silica shell for stability and to allow for functionalization with both a marker and targeting agent. The marker chosen was Rhodamine B, whereas the targeting agent was a peptide called c(RGDfK)—small and cyclical in structure that targets angiogenic cancers through α_V_β_3_ (a vitronectin receptor) binding. The determined sets of relaxivity rates for *r*_1_ and *r*_2_ on a 3 T scanner for the BDC and BTC nanorods alone were *r*_1_ = 5.5 and 7.8 mM^−1^ s^−1^ and *r*_2_ = 80.0 and 70.8 mM^−1^ s^−1^, respectively, on a per Mn basis. A decrease in MR relaxivities was observed from the BTC silica-coated nanorods to the BTC nanorods, again attributed to the reduced influence of the Mn metal centres on the surrounding water molecules. These particles, after the authors concluded modest *r*_1_ values, were destined to be vehicles for the high delivery of Mn^2+^ ions, previously found to exhibit high *r*_1_ values inside the cells due to the ions' binding to protein [137]. The *t*_1/2_ of the coated versus uncoated MOFs showed an increase of 4 h, which was reduced when run in PBS. However, even the shortened time was deemed substantial for targeting to occur and the T_1_-weighted contrast enhancement was measurable. Human colon cancer cells (HT-29) were used to determine MRI efficacy. The confocal images seemed to support the conclusion that the c(RGDfK) peptide was successful in increasing uptake of the particle through expression of Rhodamine B inside the cells. However, the *in vitro* MR images were not of high enough quality to definitely claim selective uptake through higher signal in T_1_-weighted imaging. An *in vivo* mouse study showed more substantial changes in imagery, portraying T_1_-weighted images of the midbody of a mouse model before contrast, 13 min, and 65 min after the injection of the particles at 10 µmol kg^−1^ Mn dose, which were attributed to the release of Mn^2+^ ions from the nanoparticles.

**Figure 7.**
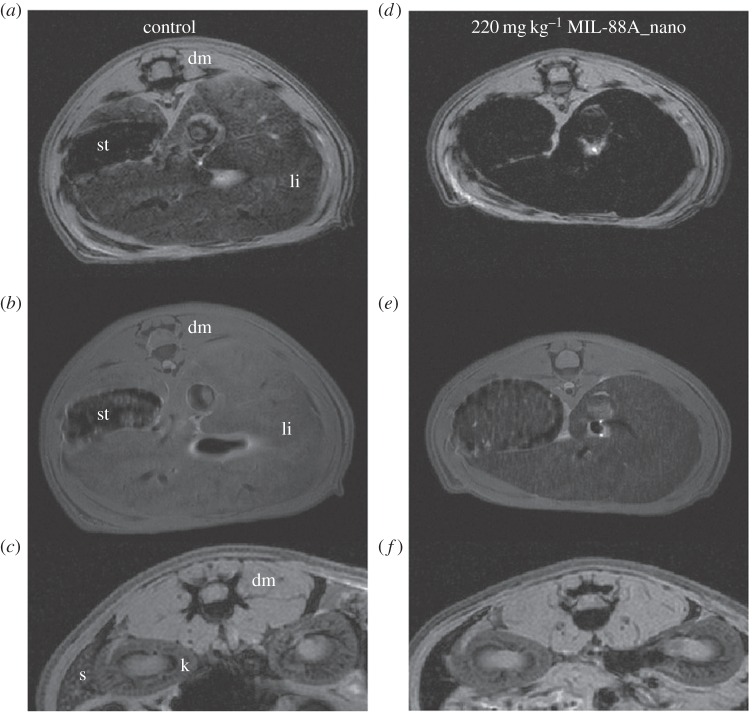
The images were acquired with gradient echo (*a*,*c*,*d*,*f*) or spin echo (*b*,*e*) sequence of control rats (*a*–*c*) and rats injected with 220 mg kg^−1^ MIL-88A (*d*–*f*), in liver (*a*,*b*,*d*,*e*) and spleen (*c*,*f*) regions; 30 min after injection, product effect is observable on the liver and spleen. dm, dorsal muscle; *k*, kidney; li, liver; s, spleen; st, stomach [113].

Horcajada *et al.* [113] were able to successfully demonstrate iron-based MOF MRI contrast agent potential through *in vivo* experimentation. Non-toxic iron(III) carboxylate MOFs (with specific focus on MIL-88A) were synthesized with adaptations to produce them at the nanoscale. All the MIL-series MOFs formulated were loaded with various challenging anti-cancer or anti-viral drugs, but the unloaded particles were used for consideration of contrast imaging potential. It was determined by Mössbauer spectroscopy that the MOF itself, and not the degradation products, were acting as the contrast agents [113], unlike the previously mentioned literature [41,126]. Wistar rats were injected with suspensions of MIL-88A particles of 220, 44 and 22 mg kg^−1^ and measured 30 min after injection. The gradient echo and spin-echo sequences showed notable differences between the liver versus the dorsal muscle and stomach in figure 7*a*,*b*,*d*,*e*, and spleen versus the dorsal muscle and kidney in figure 7*c*,*f*. The differences can best be described as a darkening of the organ. Note that lightened organ colour was restored to that of the MRI of the untreated animals three months after injection. The effect that the MOFs were having on the system correlates with their relaxivities, measured at 9.4 T, reported at *r*_2_ = 56 and 95 s^−1^ mM^−1^ for MIL-88A and PEGylated MIL-88A, respectively. The explanation for the differences in *r*_2_ values was twofold: by forming a superficial ‘brush’ on the MOF surface, the PEG coating could both increase the size of individual nanoparticles and also prevent their aggregation [128]. These effects would allow for more metal-coordinated water, following the rationale that higher quantity and mobility of metal-coordinated water gives a higher relaxivity. Additionally, cytotoxicity analysis of the *in vivo* system showed a slight increase in liver and spleen weights. This was attributed to rapid sequestration by the reticuloendothelial organs of the non-PEGylated MOFs. Overall, the iron-based cores possess good relaxivities that demonstrate potential use as MRI contrast agents.

## 4. Conclusion

In this review, we have discussed the use of MOFs as quantitative and qualitative biosensors. Biosensing via the harnessing of luminescence has enabled quantitative detection of various compounds including DPA, ssDNA and dsDNA, as well as their use as biomimetic catalysts. The importance of specificity and selectivity of MOFs is exceptionally important in ‘turn-off’ platforms in which unintended targets could quench fluorescence and lead to false readings. ‘Turn-on’ sensors are, therefore, generally preferred in luminescence-based sensing platforms, as only selective binding will lead to a signal. We believe that the furthering of the field will develop and explore a new generation of substrates and expand the library of existing MOF biosensors.

Additionally, this review has shown the use of MOFs as contrast agents in both fluorescence imaging and MRI. Incorporation of targeting groups through simple post-surface modification allows for greater binding to and uptake of the MOF particle by the intended cell target. Illumination solely of the region of interest, by using a MOF's inherent fluorescence, can achieve better diagnosis. This diagnosis can then be coupled to therapeutic delivery, creating a multimodal imaging system. However, the lack of literature on MRI with non-toxic metal cluster MOFs demonstrates the need for progression in the field and consideration for key biological properties, such as cytotoxicity and clearance times. Additionally, the lack of variation of MOFs being used shows that there is room to develop novel MOFs as highly efficient biosensing platforms.

Nevertheless, MOFs have shown promising characteristics to be potential materials in the future of biosensing. Their ideal properties, especially their flexibility and tuneable synthesis, allow for the smart design of frameworks that could pave the way towards creation of sensors with unique molecular specificity and/or precise targeting for further optical or MR imaging.
